# Phage therapy treatment of the coral pathogen *Vibrio coralliilyticus*

**DOI:** 10.1002/mbo3.52

**Published:** 2012-12-14

**Authors:** Yossi Cohen, F Joseph Pollock, Eugene Rosenberg, David G Bourne

**Affiliations:** 1 Department of Molecular Microbiology and Biotechnology, Tel-Aviv UniversityTel Aviv, 69978, Israel; 2 Australian Institute of Marine Science (AIMS)PMB3, Townsville MC, Townsville, Australia; 3 ARC Centre of Excellence for Coral Reef Studies, School of Marine and Tropical Biology, James Cook UniversityTownsville, Australia

**Keywords:** Coral disease, coral juveniles, phage therapy, *Vibrio coralliilyticus*, white syndrome

## Abstract

*Vibrio coralliilyticus* is an important coral pathogen demonstrated to cause disease outbreaks worldwide. This study investigated the feasibility of applying bacteriophage therapy to treat the coral pathogen *V. coralliilyticus*. A specific bacteriophage for *V. coralliilyticus* strain P1 (LMG23696), referred to here as bacteriophage YC, was isolated from the seawater above corals at Nelly Bay, Magnetic Island, central Great Barrier Reef (GBR), the same location where the bacterium was first isolated. Bacteriophage YC was shown to be a lytic phage belonging to the Myoviridae family, with a rapid replication rate, high burst size, and high affinity to its host. By infecting its host bacterium, bacteriophage YC was able to prevent bacterial-induced photosystem inhibition in pure cultures of *Symbiodinium*, the photosymbiont partner of coral and a target for virulence factors produced by the bacterial pathogen. Phage therapy experiments using coral juveniles in microtiter plates as a model system revealed that bacteriophage YC was able to prevent *V. coralliilyticus*-induced photoinactivation and tissue lysis. These results demonstrate that bacteriophage YC has the potential to treat coral disease outbreaks caused by the bacterial pathogen *V. coralliilyticus*, making it a good candidate for phage therapy treatment of coral disease.

## Introduction

Coral reefs are highly diverse and dynamic ecosystems, often compared with tropical rainforests in their biological complexity (Reaka-Kudla 1997). They are also one of the most productive ecosystems on earth, generating structural habitat and nutrition for a wide variety of species, including vertebrates, invertebrates, algae, and microorganisms (Hoegh-Guldberg 1999). However, assessments over recent decades have documented dramatic deterioration in coral reef health with 1–2% annual declines of coral cover over broad areas of the Indo-Pacific and greater than 50% cover coral loss of in some regions (Bruno and Selig 2007). Factors contributing to these declines include poor water quality associated with increased sedimentation and nutrients from disturbed coastlines, over exploitation of key marine species, destructive fishing, and pollution (Hughes et al. 2003). Moreover, these anthropogenic impacts, combined with rising seawater temperatures linked to climate change and increased greenhouse gases, have been correlated with the spread of coral diseases (Knowlton 2001; Bourne et al. 2009). The ecological consequences of widespread disease outbreaks include lowered coral reproduction and growth rates, altered community structures, and decreased abundance of other reef-associated organisms (Loya et al. 2001).

Although more than 20 coral diseases have been described, causative agents have been isolated for only a few diseases (Rosenberg et al. 2007; Bourne et al. 2009). Bacteria, viruses, protozoa, and fungi have all been shown to cause lesions on corals, often leaving the skeleton free of any live tissue. *Vibrio coralliilyticus* is an emerging coral pathogen that has been associated with coral disease from geographically distinct global regions. First isolated from diseased and bleaching corals off the coast of Zanzibar (Ben-Haim et al. 2003a,b), this species has also been implicated in white syndrome disease outbreaks in the Indo-Pacific (Sussman et al. 2008). Recent studies have demonstrated the ability of *V. coralliilyticus* strains to also cause disease in soft coral species, oyster larvae, and bivalves larvae (Hada et al. 1984; Austin et al. 2005; Bally and Garrabou 2007) in the other parts of the world. In hard corals, infection by *V. coralliilyticus* results in paling of coral tissues due to loss of *Symbiodinium* cells from the coenosarc tissue (the live tissue between polyps) and subsequent tissue loss leaving behind bare, white skeleton (Sussman et al. 2008, 2009). *Vibrio coralliilyticus* was found to have high proteolytic activity, producing a Zn-metalloprotease protein that plays an important role in the cleavage of connective tissue and other cellular perturbations. Addition of *V. coralliilyticus* supernatants to coral juveniles causes not only inhibition of photosynthetic activity, as with the in vitro *Symbiodinium* cells, but also loss of *Symbiodinium* cells from the coral juveniles and rapid onset of tissue lesions followed by complete mortality of the juvenile colony (Sussman et al. 2009). *Vibrio coralliilyticus* is an important model coral bacterial pathogen, with information accumulating on its virulence capacity, genome structure (De Santos et al. 2011), and protein expression potential (Kimes et al. 2012).

The worldwide decline of coral reefs necessitates the development of tools and strategies for the control and treatment of coral diseases. Coral diseases caused by bacterial infection cannot be treated with antibiotics because of the general effect of antibiotics on bacteria and the potential dangers of selection for antibiotic-resistant strains (Parisien et al. 2007). Corals also do not have an adaptive immune system (Nair et al. 2005) and therefore cannot be immunized to prevent infection. Phage therapy represents a promising alternative strategy for treatment of disease outbreaks (Housby and Mann 2009). Recent studies have reported successful closed system phage therapy trials on *Pocillopora damicornis* coral undergoing bleaching and tissue lysis caused by *V. coralliilyticus* strain YB1 and white-plague-like disease of *Favia favus* caused by *Thalassomonas loyaeana* (Efrony et al. 2007, 2009). While these studies demonstrate the potential for phage therapy in treating coral diseases in the Red Sea, similar investigations have not been conducted in other regions of the world. The study presented here investigates the potential of phage therapy for treatment of coral disease on Australia's Great Barrier Reef. A phage specifically infecting *V. coralliilyticus* strain P1 was isolated from the waters of Nelly Bay, Magnetic Island, and tested in model systems, including cultured coral endosymbionts and coral juveniles.

## Materials and Methods

### Bacterial strain, growth media, and culture conditions

The bacterial strain used in this study was *V. coralliilyticus* P1 (LMG23696), previously isolated from diseased *Montipora aequituberculata* coral colonies at Magnetic Island off the coast from Townsville, within the central section of the Great Barrier Reef Marine Park (Sussman et al. 2008). Strain P1 was maintained on MB agar plates containing: 1.8% Difco Marine Broth (Difco, Detroit, MI), 0.9% NaCl, and 1.8% Difco Bacto agar. This strain was grown routinely in liquid marine broth tryptone (MBT) medium containing 1.8% Difco Marine Broth, 0.9% NaCl, and 0.45% Difco Bacto Tryptone. Liquid cultures of strain P1 were prepared from single bacterial colonies inoculated into 50-mL Falcon tubes containing 10 mL of MBT and then incubated at 28°C in a shaking incubator at 190 revolutions per minute (rpm) for 24 h.

### Bacteriophage isolation and preparation of high-titer phage stocks

Three liters of seawater were collected from Nelly Bay, Magnetic Island, the same location where strain P1 was originally isolated. The seawater was treated with 0.5% chloroform and filtered successively through 0.8-μm and 0.22-μm membrane filters (Millipore, Bedford, MA). A series of enrichments was performed, first by adding 1 mL of bacteria (10^9^ cells) and 10 mL of MBT into 100 mL of the filtrate and incubating for 24 h in a shaking incubator at 28°C and 210 rpm. This first enrichment was centrifuged at 5250*g* and then filtered through 0.22-μm syringe filters (Millex, Millipore). The second enrichment was performed by adding 1 mL of the first enrichment to 40 mL of MBT and 1 mL of bacteria (10^9^ cells) and incubating for 24 h with shaking at 210 rpm. A 2-mL aliquot of the resulting culture was centrifuged at 5250*g* and filtered through a 0.22-μm syringe filter. Both enrichments were plated according to the soft agar overlay technique described by Adams (1959) using strain P1 as a bacterial lawn for plaque formation. Several rounds of plaque purifications were performed to ensure a pure phage stock. High-titer phage lysates were prepared by infecting strain P1 (approximately 10^9^ cells) with isolated plaques. After 5 h, the culture became clear, and following centrifugation at 5250*g* and 0.22-μm syringe filtration, the phage lysate was free of bacterial contaminants. The resulting purified phage was termed bacteriophage YC.

### Electron microscopy

Morphology-based classification of bacteriophage YC was determined using transmission electron microscopy (TEM). The filtered high-titer phage lysate (10^9^ pfu/mL) was negatively stained with 1% uranyl acetate and examined by JOEL 840A electron microscope at 80 kV as described by Efrony et al. (2007).

### Phage nucleic acid purification

Bacteriophage YC lysate (10^9^ pfu/mL) was treated with DNase I and RNase A at 37°C for 15 min to remove any contaminating free nucleic acids. The phage was then concentrated and precipitated using 20% polyethylene glycol 8000 (PEG 8000) containing 2 mol/L NaCl, EDTA (to a final concentration of 20 mmol/L), and 0.5% SDS followed by incubation at 68°C for 5 min. The resulting phage nucleic acid was purified using a phenol/chloroform, chloroform, and isopropanol extraction and precipitated with 70% cold ethanol (Sambrook et al. 1989). Genome size of bacteriophage YC was determined with 1% agarose gel electrophoresis, using two DNA ladders as markers: Gene Ruler™ 1-kb DNA ladder and Gene Ruler™ 10-kb DNA ladder (Fermentas Inc., Hanover, MD).

### One-step growth curve

One-step burst size experiments were performed in MBT medium by adding 10^7^ bacteria and 10^5^ bacteriophage (multiplicity of infection [M.O.I.] = 0.01). Exponentially growing cultures of strain P1 were infected with bacteriophage YC and incubated for 5 min at 30°C to allow the phage to adsorb onto the bacteria. The cultures were than centrifuged at 9000 rpm and the pellet resuspended in 1-mL MBT to eliminate free phage. Samples were plated periodically using the soft agar overlay technique, and plaques were counted after overnight incubation at 30°C.

### Bacteriophage adsorption kinetics experiments

Strain P1 was grown overnight to a culture density of 10^9^ cells/mL, the bacteria were pelleted by centrifugation at 5250*g*, and the pellet was washed twice with filtered artificial seawater (FASW). An adsorption kinetics experiment was then performed by adding 10^4^ phage/mL to bacteria diluted in FASW to three different bacterial concentrations: 10^5^ P1/mL, 10^4^ P1/mL, and 10^3^ P1/mL. The bacteria and phage mixture were introduced into 20 mL ASW to resemble the natural seawater environment. In flasks containing 10^5^ P1/mL and 10^4^ phage/mL, samples were taken every 10 min for 60 min; in flasks containing 10^4^ P1/mL and 10^4^ phage/mL, samples were taken every 20 min for 120 min, and in flasks containing 10^3^ P1/mL and 10^4^ phage/mL, samples were taken every 30 min for 180 min. Flasks were placed in a shaking incubator at 28°C and 190 rpm. Samples (0.3 mL) were removed from flasks, centrifuged for 4 min at 9000 rpm to remove adsorbed phages, and measurements were performed by direct plating with the soft agar overlay technique. Adsorption rate constants of phage onto bacteria were calculated by the formula: d*p*/d*t* = *K*[*P*][*B*], where d*P*/d*t* is the rate of adsorption of phage onto bacteria (phage/mL/min), [*P*] and [*B*] are concentrations of phage and bacteria, respectively, and *K* is the adsorption constant.

### *Symbiodinium* culture preparation

*Symbiodinium* growth medium was prepared by combining a modified F/2 dinoflagellate media and Erdschreiber media (Guillard and Ryther 1962). Seawater was supplemented with 4 mg/L Na_2_HPO_4_, 1 g/L NaNO_3_, 1 mL/L from a 1000× concentrated A_5_+CO micronutrient solution (described by Sussman et al. 2009), 2.5 mg/L GeO_2_, 80 mg/L G-penicillin, 80 mg/L streptomycin, 40 mg/L amphotericin, 0.4 mg/L thiamine HCl, 2 μg/L biotin, and 2 μg/L vitamin B12 (cyanocobalamin). The growth medium was 0.22 μm filtered and stored at 4°C in the dark. Before use, the 0.22-μm filtration was repeated. *Symbiodinium* cultures grown in F2 medium were inoculated into sterile 96-well plates (100 μL per well), covered and sealed. Plates were incubated at 26°C under 12 h:12 h light:dark irradiance (120 pmol photons/m^2^/sec). Cells were inspected daily and contaminated plates were discarded. Before experimental exposure, *Symbiodinium* cells were quantified (*n* = 10) using a Neubauer hemocytometer and adjusted to a uniform concentration (1 × 10^6^ cells/mL) by adding F/2 medium, and transferred into new sterile 96-well microtiter plates (100 μL per well). The microtiter plates were incubated at 26°C under 12 h light:12 h dark photoperiods (120 pmol photons/m^2^/sec) and before experimental exposure temperature was raised to 28°C at a rate of 1°C per day.

### Quantum yield measurements and PSII inactivation rates

Ten-milliliter cultures of P1 bacteria were grown for 20 h at 28°C and 190 rpm to reach a concentration of 10^9^ bacteria mL^−1^. A separate culture of strain P1 (10 mL) was grown for 20 h with 10^4^ phage YC added after 4 h of bacterial growth. Cultures were centrifuged at 5250*g* for 10 min and subsequently filtered through 0.22-μm syringe filters. Hundred microliters of clarified supernatant was then added to each microtiter well (each supernatant treatment was conducted in triplicate) and exposed to a maxi imaging pulse amplitude modulation (MAXI iPAM) fluorometer saturation light pulse (Gain = 1–2, Intensity = 1–2, Saturation pulse = 7) at 0, 1, 2, and 4 h followed by 4-h intervals up to 24 h. The *Symbiodinium* cells were dark adapted for 30 min prior to each measurement to obtain dark adapted quantum yields (QYs) (*F*_v_/*F*_m_), which were calculated using the formula, *F*_v_/*F*_m_ = (*F*_m_ − *F*_0_)/*F*_m_ (*F*_m_ = maximum fluorescent yield, *F*_0_ = dark fluorescent yield). PSII inactivation rates were calculated as proportions from the control wells containing only *Symbiodinium* cells only ([QY_control_ − QY_treatment_]/QY_control_) × 100).

### Effect of different concentrations of strain P1 with Bacteriophage YC on photoinactivation rates of coral endosymbionts

Bacteria were grown in 50-mL Falcon tubes for 20 h at 28°C and 190 rpm in a shaking incubator to reach a concentration of 10^9^ cells/mL before being diluted with FASW to reach concentrations of 10^5^–10^9^ cells/mL (verified by plating on TCBS [thiosulfate-citrate-bile salts-sucrose] agar plates). For each concentration, two separate tubes were prepared, one with addition of 10^4^ phage/mL and another with no phage addition. All tubes were placed in a shaking incubator at 28°C and 190 rpm for 5 h and samples were then centrifuged and filtered through a 0.22-μm filter to obtain bacteria-free supernatants. From each sample, 100 μL of derived supernatant was added to wells of a 96-well microtiter plate containing 100 μL of 10^6^ mL^−1^ *Symbiodinium* cells. The plate was exposed to a maxi iPAM fluorometer saturation light pulse (Gain = 1–2, Intensity = 1–2, Saturation pulse = 7) at 0, 1, 2, and 4 h followed by 4-h time intervals until 36 h. PSII inactivation rates were calculated as proportions from quantum yield measurements by the MAXI iPAM fluorometer.

### Collection and maintenance of *Acropora millepora* coral juveniles

*Acropora millepora* coral juveniles were raised from larvae as described by Abrego et al. (2008) and Littman et al. (2010). Briefly, *A. millepora* parental colonies were collected from Cattle Bay, Orpheus Island, prior to spawning in November 2010. Following spawning, gametes were reared at Orpheus Island Research Station to produce larvae. After settlement and infection with clade D algal symbiont, the coral juveniles were returned to the waters off of Pelorus Island. Juveniles attached to terra cotta tiles were placed on steel rods and suspended horizontally between pairs of metal star-pickets on the reef flat on the west side of Pelorus Island where they were allowed to grow for 6 months. The terracotta tile racks were removed from the reef by snorkeling and carried in large containers to the Australian Institute of Marine Science (AIMS) where they were placed in outdoor aquaria facilities with 5 μm filtered flow through seawater for 1 week to allow acclimatization. After 1 week, juveniles were removed from the tiles using a microscope, scalpel, and tweezers and placed into individual wells in a 12-well plate with 5 mL of FASW. All plates containing juveniles were incubated at 24°C under 12 h light:12 h dark photoperiods with irradiance of 120 pmol/m^2^/sec. A health assessment of the juveniles was performed every 2 days, checking the pigmentation and juvenile's extracted tentacles. Juveniles displaying signs of stress were removed from the plates. In addition, FASW was replaced every 2 days with fresh 5 mL of FASW. After 5 days, the temperature was elevated by 2°C from 24°C to 26°C and 5 days after the temperature was again increased to 28°C.

### Phage therapy experiment on *A. millepora* coral juveniles

Three individual colonies of strain P1 were inoculated into separate culture tubes of MBT medium and FASW at a 1:1 ratio (5 mL MBT: 5 mL FASW) and incubated for 24 h in a shaking incubator at 190 rpm with phage YC (10^4^ mL^−1^) added at different time points of bacterial growth (2, 8, and 18 h). Overnight cultures were centrifuged and filtered leaving clear, bacteria-free supernatants. To each well containing an *A. millepora* juvenile, FASW and supernatant at a 1:1 ratio (2 mL FASW: 2 mL of supernatant) was added. Interval measurements (0, 1, 3, 6, 9, and 12 h) of quantum yield and PSII inactivation rate were determined using a MAXI iPAM fluorometer, and the change in the juveniles' morphology (0, 1, 3, 6, and 9 h) was observed using a dissecting microscope and digital camera. Controls consisted of juveniles exposed only to the addition of FASW.

## Results

### Phage isolation and characterization

**Figure 1:**
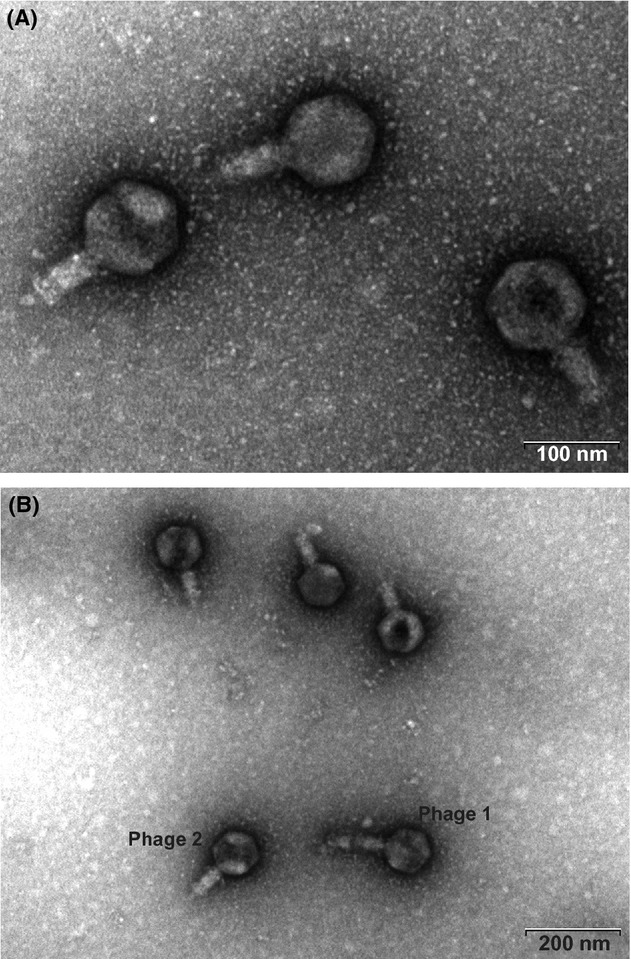
Transmission electron microscope (TEM) micrographs of negatively stained bacteriophage YC. (A) A 100-nm scale TEM photo shows the hexagonal capsid head of phage YC and the contractile tale. (B) A 200-nm scale TEM photo shows phage YC genomic DNA inside its capsid. Phage 1 shows the noncontractile tail outline. Phage 2 shows the contractile tail outline that is 50% shorter than the noncontractile mode. According to its morphology, phage YC is a member of the Myoviridae family.

**Figure 2:**
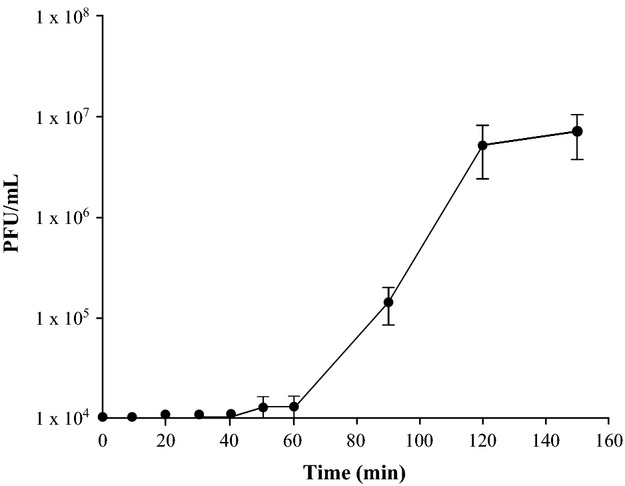
One-step growth curve and burst size of bacteriophage YC in MBT medium. Phage YC was used at multiplicity of infection (M.O.I.) of 0.01.

Bacteriophages specific for the coral pathogen *V. coralliilyticus* strain P1 were isolated from filtered and chloroform-treated seawater. Clear plaques were observed on P1 bacterial lawns grown on MA agar. A single plaque, referred to as phage YC, was selected and further purified for this study. Electron micrograph images of the YC bacteriophage were obtained from highly concentrated phage lysates (Fig. 1A and B). The phage demonstrated a nonenveloped, head–tail structure. The tail is contractile and the capsid has elongated icosahedral symmetry morphology consistent with the Myoviridae family. The genome size of the YC phage was determined by total extraction of DNA and comparison with standards on a 1% agarose gel, which showed that the phage has a small genome of 11 kb. The phage nucleic acid was sensitive to DNAse but not RNAse indicating it is a DNA phage. The packaged viral genomic DNA can be observed in the TEM micrograph inside the capsid head that has a 90-nm diameter (Fig. 1A). The phage tail is 80 nm which is characteristic of the size of the Myoviridae family of viruses (Fig. 1B). The latent phase for bacteriophage lysis of the P1 strain was 60 min as determined by a one-step burst size experiment (Fig. 2). The experiment was conducted in nutrient-rich MBT medium to partially mimic the nutrient-rich environment that a bacterium may experience in the coral mucus. Absorption of the phage to the bacterium is rapid and occurred within 5 min at 30°C. The burst size was 500 phage particles per infected bacterial cell. The adsorption rate of bacteriophage YC in low nutrient medium was determined in an attempt to mimic the behavior and efficiency of the phage in seawater samples. For concentrations of 10^5^ P1/mL and 10^4^ phage/mL in low nutrient media, an adsorption of 70% was observed after 50 min (Table 1). At concentrations of 10^4^ P1 and 10^4^ phage/mL, adsorption of 45% after 60 min was observed. For the low bacterial concentration treatment of 10^3^ P1 and 10^4^ phage/mL, adsorption of 41% was observed after 60 min. The average *K* value for all three treatments was 4.8 × 10^−5^ ± 2.6 × 10^−6^ phage/mL/min (SE).

**Table 1:** Phage YC Adsorption Kinetics onto P1 bacteria

**Concentration of bacteria and phage**	10^5^ P1 + 10^4^ phage YC	10^4^ P1 + 10^4^ phage YC	10^3^ P1 + 10^4^ phage YC
**Adsorption in time**	70% in 50 min	45% in 60 min	41% in 60 min
**Adsorption rate (*K*)**	Average *K* value for the experiment = 4.8 × 10^−5^ ± 2.61 × 10^−6^ phage/mL/min		

### Effect of bacteriophage YC on *V. coralliilyticus-*induced *Symbiodinium* photosynthesis inhibition

**Figure 3:**
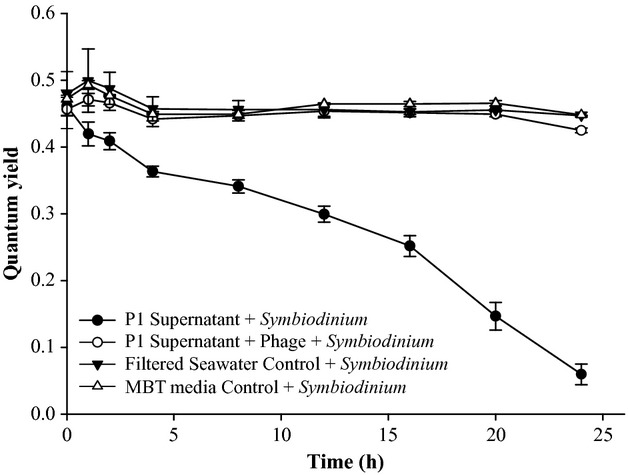
Photosynthetic quantum yield measurements of *Symbiodinium* after exposure to *Vibrio coralliilyticus* P1 bacterial supernatants with and without bacteriophage YC treatment.

*Symbiodinium* cultures of clade D treated with P1 bacterial supernatant showed a decrease in the photosynthetic activity after the P1 supernatant was added. For example, after 12 h, the quantum yield of *Symbiodinium* decreased by 40%, and by 24 h, a 90% reduction in the *Symbiodinium* quantum yield was observed (Fig. 3). In contrast, the quantum yield of *Symbiodinium* exposed to the supernatant of P1 bacteria infected with bacteriophage YC showed no change in their quantum yield values and resembled the controls (Fig. 3).

**Figure 4:**
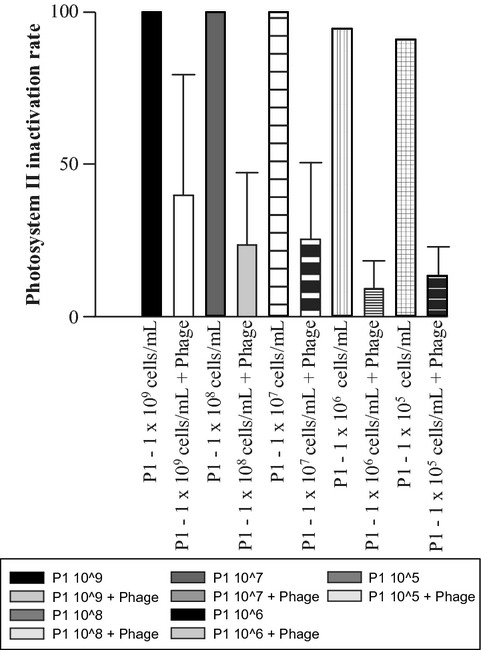
Photoinactivation of *Symbiodinium* cells through 36-h exposure to supernatant from different starting concentrations of *Vibrio coralliilyticus* P1 (10^9^, 10^8^, 10^7^,10^6^, 10^5^ cells/mL), and to supernatant from the same starting concentrations of bacteria that were infected with phage YC (10^4^ pfu/mL).

The PSII photoinactivation rate of the corals' *Symbiodinium* endosymbionts exposed to different concentrations of *V. coralliilyticus* strain P1 supernatant was directly related to the concentration of supernatant added. All concentrations of bacterial supernatant caused strong photoinactivation (>90%), though higher concentrations of P1 supernatant demonstrated higher photoinactivation rates over the 36-h experiment. For example, supernatant extracted from 10^9^ P1 cells resulted in a 100% inactivation rate while supernatant extracted from 10^5^ P1 cells caused 91.1% of inactivation rate after 36 h (Fig. 4). When 10^4^ phages were added to each concentration of P1, photoinactivation rates of the *Symbiodinium* endosymbionts were significantly lower (*P* < 0.005). For example, bacterial supernatants extracted from 10^9^ P1 cells with added phage caused only 50% photoinactivation of *Symbiodinium* cells. Photoinactivation rates were even lower (only 9.2%) when *Symbiodinium* cells were exposed to supernatant from 10^6^ P1 cells with phage addition. In general, the extent of photoinactivation was lower when phage was added to more dilute bacterial cultures.

### Phage therapy of *A. millepora* coral juveniles

**Figure 5:**
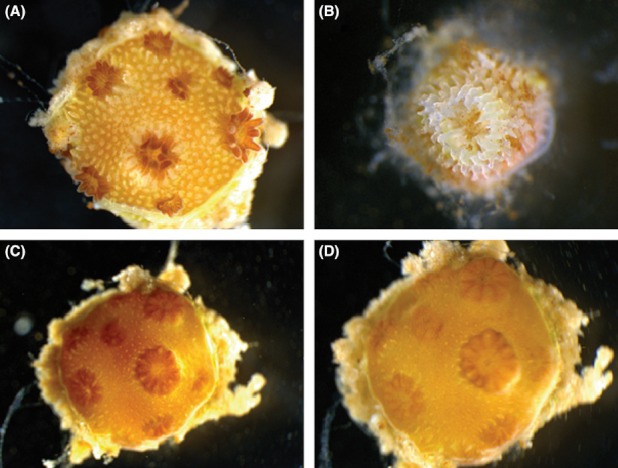
Dissecting microscopy photos of *Acropora millepora* coral juveniles after treatment with: (A) FASW only (i.e. negative control) at T = 9 h, (B) *Vibrio coralliilyticus* P1 supernatant at T = 9 h, (C) Supernatant extracted from P1 that grew in the presence of bacteriophage YC at T = 0 h, and (D) Supernatant extracted from P1 that grew in the presence of bacteriophage YC at T = 9 h.

**Figure 6:**
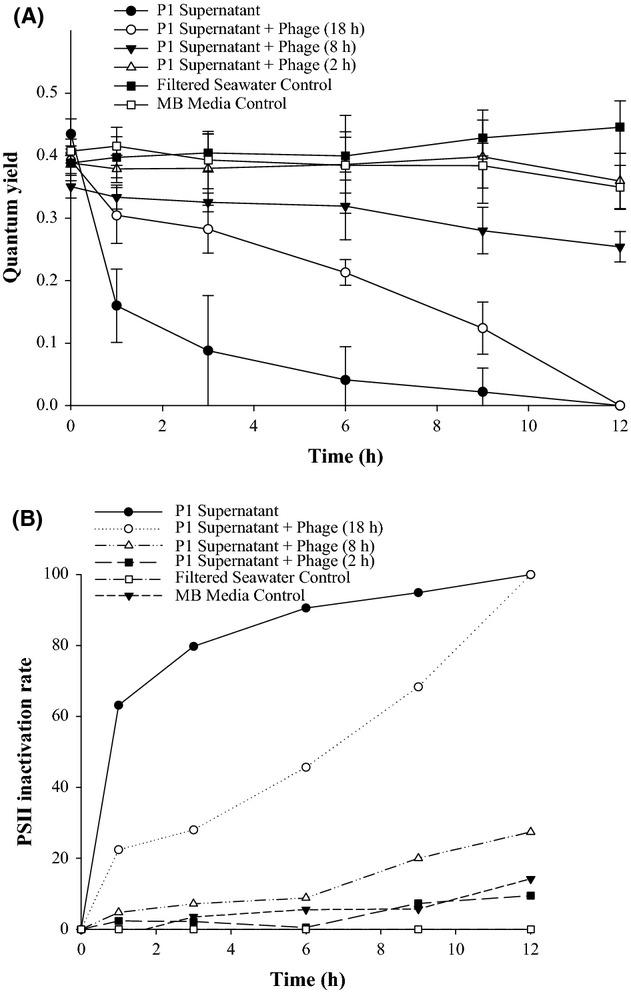
(A) Maxi imaging pulse amplitude modulation (PAM) quantum yield measurement of *Acropora millepora* coral juveniles after treatment with P1 supernatant and P1 supernatant with phage adding after 2, 8, and 18 h. Measurements were taken at time points of 0, 1, 3, 6, 9, and 12 h. Before each measurement, juveniles were dark adapted for 30 min. (B) Maxi imaging PAM *Symbiodinium* PSII inactivation rates after treatment with P1 supernatant and P1 supernatant with phage adding after 2, 8, and 18 h. PSII inactivation was calculated as a proportion from the quantum yield measurement of *A. millepora* coral juveniles.

Phage therapy of *A. millepora* coral juveniles, measured indirectly by inhibition of coral photosynthesis, was performed to examine phage treatment efficiency on live coral. Addition of *V. coralliilyticus* P1 supernatant to the wells of microtiter plates holding individual 6-month-old coral juveniles resulted in an immediate visible response. Images derived from a dissecting microscope and digital camera showed the coral's polyps immediately retract and after 1 h *Symbiodinium* cells were observed to be expelled from the coral tissue. After 3 h, degradation of the coenosarc tissue (the tissue between the polyps) was observed. After 6 h, tissue lesions appeared and after 9 h only skeleton remained (Fig. 5). The iPAM measurements showed a rapid decline in the coral's quantum yield and after 6 h of incubation, low values of 0.041 (compared with control values of 0.4) were recorded while after 12-h quantum yield measurements had fallen to zero (Fig. 6A). Calculations of PSII inactivation rates showed a complete inactivation of the reaction center after 9 h (Fig. 6B). Supernatant derived from P1 bacteria with added phage after 2 h of bacterial growth demonstrated very different effects on the *A. millepora* juveniles. Dissecting microscope images showed no change in the appearance of the corals and no signs of *Symbiodinium* cells expulsion during the entire 12-h experiment. iPAM measurements showed no decline in coral quantum yields, with a value of 0.388 (±0.02 SE) recorded at *T* = 0 and values of 0.398 (±0.05 SE) and 0.360 (±0.04 SE) recorded after 6 h and 12 h of the experiment, respectively. A low inactivation rate of 7% was observed after 12 h. Assays on *A. millepora* juveniles using supernatant derived from P1 bacteria with phage added after 8 h of bacterial growth showed a similar effect to the control corals treated with the P1 supernatant without added phage. After 3 h of supernatant exposure, *Symbiodinium* cells were observed to be expelled from the coral tissue, and after 6 h distinct tissue lesions were observed on the juveniles resulting in exposed coral skeleton free of live coral tissue. iPAM measurements showed a slower decline in the quantum yield rates from 0.350 (±0.01 SE) at *T* = 0 to 0.254 (±0.02 SE) after 12 h. PSII reaction centers demonstrated an inactivation rate of 20% after 9 h. The effect on the coral juveniles of supernatant derived from P1 grown for 18 h before exposure to the phage was similar to P1 supernatants with no phage addition and P1 supernatant with phage added 8 h after growth. Tissue lesions were observed after 6 h and exposed coral skeleton observed after 9 h of exposure. Although the decline in the quantum yield by this treatment was more moderate than the decline in the P1 supernatant treatment, iPAM results showed a decrease in the quantum yield of the treated juveniles, with a rate of zero recorded after 12 h.

## Discussion

Coral disease outbreaks pose a serious risk to reef ecosystems through the destruction of reef-building corals. Robust and tangible reef management practices need to be developed to prevent coral disease outbreaks and preserve reefs currently subjected to increased anthropogenic stress and high prevalence of disease. Phage therapy, which involves the application of a virus (or viruses) that specifically kill an identified bacterial pathogen, represents one potential management tool that can be applied at reef-wide scales to manage disease outbreaks. Previous studies have identified and characterized phages specific to disease pathogens in the Gulf of Eilat in the Red Sea. This study expanded the research on phage therapy to a different geographic area, by isolating and testing the first coral pathogen-specific phage outside of the Red Sea, and examined the physiological response of *V. coralliilyticus* strain P1, previously identified as the causative agent of the white syndrome disease in *Montipora auquiberculata* corals (Sussman et al. 2008). This study also adapted models for coral health, including coral endosymbionts (*Symbiodinium*) and reared coral juveniles of *A. millepora,* to observe the effects of treatment of the coral pathogen with a specific lytic bacteriophage.

In natural environments, phages and their bacterial hosts maintain equilibrium. Populations of bacteria that bloom are often controlled by phage infection which subsequently reduces their numbers, the “kill the winner” theory (Thingstad 2000). Therefore, in natural ecosystems, wherever bacteria can be isolated, a specific phage can also generally be found (Chibani-Chennoufi et al. 2004; Stenholm et al. 2008). In this study, a bacteriophage specific to the identified coral pathogen *V. coralliilyticus* strain P1 was successfully isolated from waters of Nelly Bay, Magnetic Island. This was the location where the *Vibrio* pathogen was first isolated from diseased corals (Sussman et al. 2008). Phage YC showed high specificity to its host and was not shown to infect other strains of *V. coralliilyticus*, including strains P2 and P3 (Isolated by Sussman et al. 2008). The natural concentrations of phage in seawater can fluctuate with the season, UV light exposure, temperature, and bacterial host densities. In addition, changes in the phage concentrations might be related to a disease outbreak as it is correlated with a rise in the concentration of pathogenic bacteria. Phage YC was isolated in the summer when sea surface temperatures were 28°C and disease prevalence was relatively high (Haapkyla et al. 2010). A bacteriophage specific for *V. coralliilyticus* could only be isolated from sampled seawater treated with 0.5% chloroform. Untreated samples showed no specific enrichment for *V. coralliilyticus* specific phages indicating the YC phage may have been attached to the outside of the bacterium or in a pseudolysogenic state within the natural populations of *V. coralliilyticus*. Chloroform is effective at causing cell disruption and may have released the phage. This observation is consistent with the concept that phages would “prefer” to remain associated with the bacterium, as a survival strategy, when the host in the natural environment is at low densities (Moebus 1996). When phage YC was plated by the soft agar overlay technique after a second enrichment on nutrient-rich media, clear viable plaques were established on the MA plate. It is possible that some of the phage turned to the lytic cycle when the environment became more favorable to its host.

TEM photos of the isolated phage indicated that it belongs to the Myoviridae family based on its conserved and highly characteristic morphology. No genotypic characterization was possible as there are no taxonomic genetic markers for identification of phages (Paul and Sullivan 2005). If we rank phage YC according to the rank-abundance curve (Suttle 2005), it appears to be an r-selected strategist: small, virulent, opportunists, which replicate quickly, have short life cycles and produce many progeny.

Several studies have examined marine phage adsorption kinetics and a comparison between adsorption rates of these marine phages highlights that phage YC has a high adsorption rate (Table 2). Furthermore, the phage absorption kinetics of YC were similar to BA3 bacteriophage that was used in a previous study against white-plague-like coral disease in the Red Sea (Efrony et al. 2009). The adsorption constant (*K*) of 4.8 × 10^−5^ ± 2.6 × 10^−6^ phage/mL/min indicates that YC is capable of rapidly adsorbing onto *V. coralliilyticus* P1. No threshold value was observed for phage absorption at low bacterial concentrations of 10^3^ cells/mL in contrast to previous phage kinetic studies (Payne and Jansen 2001). The phage was also successful in binding to the target *V. coralliilyticus* P1 strain in seawater with a burst of 500 new phages when it comes into contact with a nutrient-rich area similar to coral mucus.

**Table 2:** Comparison of adsorption rates of other identified marine phages to phage YC

Phage name	Host	Adsorption kinetics rate (*K*)	Reference
Cyanophages	*Synechococcus* spp.	3.94 × 10^−9^ phage/mL/min	Suttle and Chan (1993)
AaV-1	*Aureococcus anophagefferens*	7.2 × 10^−9^ phage/mL/min	Garry et al. (1998)
MpV	*Micromonas pusilla*	1.4 × 10^−9^ phage/mL/min	Cottrell and Suttle (1995)
BA3	*Thalassomonas loyaeana*	1.0 × 10^−6^ phage/mL/min	Efrony et al. (2009)
Phage YC	*Vibrio coralliilyticus* P1 strain	4.8 × 10^−5^ ± 2.6 × 10^−6^ phage/mL/min	This study

Bacterial causative agents for a number of specific coral diseases have been previously identified (Rosenberg et al. 2007); however, little is known regarding specific bacterial virulence mechanisms that enable the lesions to progress. Studies investigating the virulence mechanisms of the coral pathogen *V. coralliilyticus* demonstrated that an expressed Zn metalloprotease caused photoinactivation of the coral photosynthetic *Symbiodinium* endosymbionts (Banin et al. 2003; Ben-Haim et al. 2003a,b; Sussman et al. 2009). Moreover, a recent study by De Santos et al. (2011) demonstrated that strain P1 has a diverse repertoire of metalloproteases that enable *V. coralliilyticus* to be potentially highly virulent. The addition of phage YC to the growth medium of the P1 strain resulted in lysis of the bacterium and therefore presumably prevented expression of the metalloprotease and subsequent inhibition of the photosystems of the *Symbiodinium* cells. *Symbiodinium* cells in the coral juvenile did not show any effect of the Zn metalloprotease on its quantum yield, when supernatant produced from P1 strain with phage added to culture after 2-h growth was dosed onto the coral. Sussman et al. (2009) demonstrated that the production rate of Zn metalloprotease is highest at bacterial densities of 10^8^–10^9^ cells/mL and that the bacterium continues to produce metalloprotease at high rates even after reaching the stationary growth phase. Addition of the phage to the bacterial cultures reduced *Symbiodinium* PSII inactivation rates rapidly even at high concentrations of bacteria (10^9^ cells/mL). This phage addition lysed the bacterial pathogen and presumably stopped Zn-metalloprotease production and further damage to *Symbiodinium* cells. The concentration of a pathogen in a diseased coral can reach a concentration of almost 10^9^ cells/mL (Israeli et al. 2001). Therefore, the isolated phage offers good potential as a treatment to stop the spread of a pathogen to other corals. When added at an early stage of disease progression, the phage has the potential to help the coral and its associated endosymbionts defend themselves against pathogen invasion.

Coral juveniles represent an excellent model system to study coral's *Symbiodinium* recruitment, coral fitness, and coral disease mechanisms (Abrego et al. 2009; Mieog et al. 2009; Sussman et al. 2009). Sussman et al. (2009) showed that coral juveniles demonstrate characteristic disease signs when exposed to bacterial supernatants of strain P1, *Symbiodinium* PSII inactivation, paling of coral tissue as a result of loss in *Symbiodinium* cells followed by formation of coral tissue lesions and subsequent mortality. The disease signs observed on the juveniles are similar to disease signs observed on the coral reef (Anthony et al. 2008) and during laboratory infection experiments (Sussman et al. 2008). In the current study, juvenile exposure experiments were successfully repeated and extended to examine the potential for bacteriophage addition to protect the health of the coral juveniles. When the phage YC was added to the P1 bacterial strain, it was able to prevent the disease signs within the juveniles.

In conclusion, this study has isolated and identified a phage effective against the coral pathogen *V. coralliilyticus*. The Myoviridae-like phage possesses properties important for effective action against the bacterial pathogen and therefore represents an agent that has potential for treatment of localized coral disease outbreaks. Further research is required, however, to examine the feasibility of such a treatment in adult corals along with the benefits and risks of phage therapy treatments in an open coral reef ecosystems.
